# A postoperative dynamic nomogram for predicting myocardial injury after noncardiac surgery in high-risk patients undergoing laparoscopic colorectal cancer resection

**DOI:** 10.1186/s12871-026-03893-x

**Published:** 2026-05-13

**Authors:** Yuanqiang Li, Wenting Jiang, Jingchen Liu

**Affiliations:** 1 Department of Anesthesiology, Nanxishan Hospital of Guangxi Zhuang Autonomous Region (The Second Peoples Hospital of Guangxi Zhuang Autonomous Region), Guilin, Guangxi 541002 China; 2 School of Nursing, Guangxi University of Chinese Medicine, Nanning, Guangxi 530200 China; 3 Department of Anesthesiology, The First Affiliated Hospital of Guangxi Medical University, 6 Shuangyong Road, Nanning, Guangxi 530021 China

**Keywords:** Colorectal cancer surgery, High-risk patients, Myocardial injury after noncardiac surgery, Dynamic nomogram, Postoperative risk stratification

## Abstract

**Background:**

Myocardial injury after noncardiac surgery (MINS) is closely associated with perioperative cardiovascular events and is a critical complication leading to poor prognosis. This study aimed to develop a nomogram model to predict the risk of MINS in high-risk patients undergoing colorectal cancer surgery, which can be applied immediately after surgery to guide postoperative troponin monitoring and individualized intervention.

**Methods:**

This study retrospectively included 358 patients at high cardiovascular risk who underwent laparoscopic colorectal cancer surgery from August 2021 to January 2023. They were assigned randomly to training and validation cohorts in a 7:3 ratio. After univariate analysis and multicollinearity assessment, multivariable logistic regression identified independent predictors associated with MINS in the training cohort, and nomogram prediction models were constructed. The nomogram model’s discrimination, calibration, and clinical validity in the training and validation cohorts were evaluated and validated.

**Results:**

Age, preoperative hemoglobin, preoperative high-sensitivity troponin T (hs‑cTnT), and the Surgical Apgar Score (SAS) were identified as independent predictors. The AUC (C‑index) of the nomogram constructed from these four predictors was 0.884 (95% CI: 0.831–0.937) and 0.853 (95% CI: 0.729–0.977) in the training and validation cohorts, respectively. Decision curve analysis indicated favorable clinical utility, with a positive net benefit across a wide range of threshold probabilities including the recommended 20% risk threshold.

**Conclusion:**

A postoperative dynamic nomogram based on preoperative and intraoperative variables provides a simple, practical tool for predicting MINS risk in high-risk patients undergoing colorectal cancer surgery. The model showed favorable discriminative ability and calibration upon internal validation, but prospective external validation is needed before clinical implementation.

**Trial registration:**

The study was registered with the Chinese Clinical Trial Registry (ChiCTR2200065961, 20/11/2022).

**Supplementary Information:**

The online version contains supplementary material available at 10.1186/s12871-026-03893-x.

## Introduction

Over the past decade, myocardial injury after noncardiac surgery (MINS) has emerged as the most prevalent and prognostically significant complication, ranking among the top three causes of mortality in patients undergoing noncardiac surgery within 30 days postoperatively [1]. MINS typically manifests within three days following surgery [1, 2]. Given that the majority of MINS cases do not present with the classic clinical symptoms of myocardial ischemia or electrocardiographic alterations, they are frequently undetected without perioperative cardiac troponin (cTn) monitoring, especially under conditions of sedation, anesthesia, and postoperative analgesia [3]. However, MINS is independently associated with the risk of death and cardiovascular events at 30 days postoperatively and during the initial year after surgery, irrespective of the presence or absence of ischemic symptoms [2, 4, 5]. In recent years, several guidelines have pointed out that cardiac troponin screening is recommended for patients with cardiovascular risk factors before surgery and 48–72 h after surgery [6–10]. However, in some settings, routine perioperative cTn screening may increase healthcare costs and pose logistical challenges, limiting its widespread adoption.

Based on clinical risk indicators, the Revised Cardiac Risk Index (RCRI) has been widely used to assess perioperative cardiac risk over the past 20 years [11, 12]. Nevertheless, the RCRI has a low correlation with MINS and does not effectively predict its occurrence [13]. To enhance the discriminatory power of the RCRI in predicting MINS, Douville et al. incorporated a polygenic risk score for coronary artery disease, integrating genetic information into the risk model [14]. Although polygenic risk scores were associated with MINS, they did not significantly improve the predictive model’s discriminatory power [14]. In addition, the American College of Surgeons National Surgical Quality Improvement Program (NSQIP) preoperative risk stratification tool had a low correlation with MINS [13]. To date, there remains a lack of effective MINS prediction tools in clinical practice that provide a clinical decision-making basis for medical staff to guide postoperative cTn monitoring and individualized interventions for high-risk patients.

Therefore, this study aimed to identify preoperative and intraoperative variables associated with MINS in high-risk patients undergoing colorectal cancer surgery, to construct and internally validate a postoperative dynamic nomogram for individualized risk prediction, and to directly compare its predictive performance with that of the traditional RCRI within the same cohort.

## Materials and methods

### Study design

This was a single-centre, retrospective cohort study. The study developed and internally validated the predictive model according to the Transparent Reporting of Individual Prognosis or Diagnosis with Multivariate Predictive Models (TRIPOD) reporting guideline statement checklist [15]. The study complied with the Declaration of Helsinki. It was approved by the Ethics Committee of the First Affiliated Hospital of Guangxi Medical University (NO.2022-K059-01), waiving the requirement for written informed consent. The study was registered with the China Clinical Trial Center (ChiCTR2200065961).

### Patient selection

High-risk patients who underwent elective laparoscopic colorectal cancer surgery at the First Affiliated Hospital of Guangxi Medical University from August 2021 to January 2023 were retrospectively included. All patients underwent preoperative high-sensitivity cardiac troponin T (hs-cTnT) testing and were screened for hs-cTnT at least twice within the first three postoperative days. High-risk patients included in this study were defined as age ≥ 65 years or age ≥ 45 years combined with a history of coronary artery disease, peripheral artery disease, or stroke [16]. Exclusion criteria were: (1) relevant case information or missing clinical data; (2) neoadjuvant therapy such as chemotherapy or immunotherapy before surgery; (3) preoperative comorbidities of sepsis, pulmonary embolism, atrial fibrillation, cardiac resuscitation, acute or chronic heart failure, or chronic renal insufficiency; and (4) a history of acute infarction, acute coronary syndromes, or unstable angina.

### Data collection and definition

Clinical data, including preoperative baseline information, laboratory test results, and patient intraoperative data, were gathered from the Hospital Information System and perioperative anaesthesia database.

Intraoperative hypertension was defined as systolic blood pressure (SBP) > 160 mmHg for at least 1 min or treatment of blood pressure with antihypertensive drugs. Intraoperative hypotension was defined as a SBP < 90 mmHg for at least 1 min or treatment with vasopressors. Although recent studies have also emphasized the importance of mean arterial pressure (MAP) < 65 mmHg, large‑scale evidence has demonstrated that SBP < 90 mmHg is comparably associated with myocardial injury and that the strength of association with myocardial injury is similar for SBP and MAP [17]. Intraoperative tachycardia was a heart rate (HR) > 100 beats/min or medication to lower HR. Intraoperative bradycardia was defined as an HR < 50 beats/min or the use of medication to raise HR. Surgical Apgar score (SAS) was calculated based on the estimated intraoperative blood loss, the lowest mean arterial pressure (MAP), and minimum HR, with a total score of 0–10 [18]. The modified surgical Apgar score (mSAS) integrates intraoperative blood transfusion into the original SAS scoring system. Specifically, when a patient received a blood transfusion during surgery, the “estimated blood loss” score was 0 [19]. According to the original definition by Lee et al. [11], the RCRI score comprises six equally weighted components: (1) high-risk surgery (intra-abdominal, intrathoracic or supra-groin vascular surgery); (2) history of ischemic heart disease; (3) history of congestive heart failure; (4) history of cerebrovascular disease (stroke or transient ischemic attack); (5) preoperative insulin therapy for diabetes; and (6) preoperative serum creatinine > 2.0 mg/dL (> 177 µmol/L).

### Study outcomes

The primary outcome was a MINS event within the first three postoperative days. The diagnostic specificity threshold for prognostic relevance of MINS was based on the 2021 American Heart Association (AHA) scientific statement [20]: a postoperative hs-cTnT concentration of 20–65 ng/L with an absolute change ≥ 5 ng/L, or a postoperative hs-cTnT > 65 ng/L, or an absolute perioperative hs-cTnT elevation > 14 ng/L (defined as peak postoperative value minus preoperative value). These thresholds have been validated as independent predictors of 30-day mortality.

### Sample size

The sample size for model development was calculated using the method proposed by Riley et al. for binary outcomes [21]. Based on 15 candidate predictor parameters, an anticipated MINS incidence of 15.1%, and a Cox-Snell *R*^2^ of 0.10 (a conservative estimate for such clinical prediction models), the minimum required sample size was 210 participants. This requirement is met by our training cohort of 251 participants.

### Statistical analysis

Statistical analyses were performed using SPSS version 26.0 (IBM Corporation, Armonk, NY, USA) and R software version 4.0.3 (R Foundation for Statistical Computing, Vienna, Austria). Normality of continuous variables was assessed using the Kolmogorov–Smirnov test. Variables with *P* > 0.05 were considered normally distributed and expressed as mean ± standard deviation (SD), and comparisons between groups were made using the independent samples t-test; otherwise, variables were expressed as median with interquartile range (IQR) and compared using the Mann–Whitney U test. Categorical variables were expressed as frequencies and percentages (%), and comparisons between two groups were made using the χ² test, Fisher’s exact test, or the rank sum test, as appropriate.

A randomized grouping code was used to divide the included patients into a training cohort and a validation cohort in a ratio of 7:3. In the training cohort, the predictors were initially screened by univariate analysis of each clinical variable one by one, and covariate diagnosis was performed for variables with *P* < 0.05 in the results of univariate analysis. Estimation was performed by the variance inflation factor (VIF) with a reference value of 10, and Variables with multicollinearity were not included in the multivariate logistic regression analysis. A nomogram was constructed based on the results of multivariate logistic regression, and the Shiny package (version 0.13.2.26) was used to develop a dynamic web-based nomogram. The nomogram was evaluated by examining the discrimination between the training and validation cohorts and by calibration. The discriminative ability of the nomogram was assessed using the area under the receiver operating characteristic curve (AUC) and the concordance index (C‑index), with 95% confidence intervals (CIs) calculated by bootstrapping with 1000 resamples. The optimal cut-off value for predicting MINS was determined by maximizing the Youden index. Calibration was evaluated by plotting calibration curves (predicted probability vs. observed frequency) and by the Hosmer–Lemeshow goodness‑of‑fit test. Clinical utility was assessed using decision curve analysis (DCA), which quantifies the net benefit of the model across a range of threshold probabilities. To facilitate clinical application, an optimal risk threshold was determined by jointly considering the Youden index, the net benefit from DCA, and the clinical trade‑off between sensitivity and specificity. Subsequently, sensitivity, specificity, positive predictive value (PPV), and negative predictive value (NPV) at this threshold were calculated from the confusion matrix for the training, validation, and pooled cohorts. The discriminative ability of the RCRI for MINS was assessed in the validation cohort using ROC analysis, and the AUC was compared with that of our nomogram using the DeLong test [22]. All statistical tests were two-sided; a *P* value < 0.05 was considered statistically significant. Supplementary materials, including the results of normality tests for key continuous variables, are available online.

## Results

### Patient characteristics

**Fig. 1:**
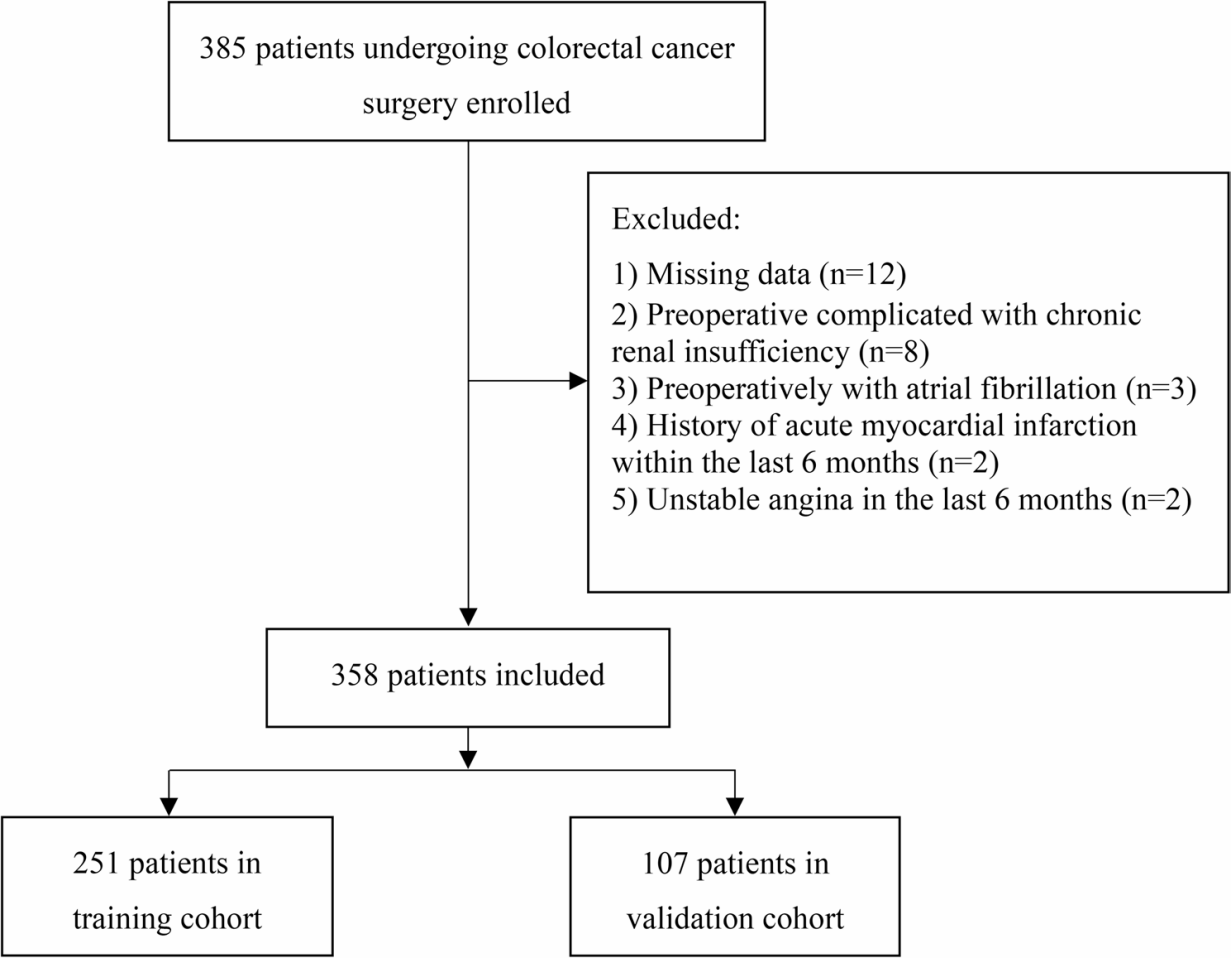
Flow diagram of patients screening and datasets partitioning

A total of 358 patients were included in the final analysis of this study, of which 251 patients were assigned to the training cohort (39 in the MINS group and 212 in the non-MINS group) and 107 patients were assigned to the validation cohort (15 in the MINS group and 92 in the non-MINS group). The study flowchart was presented in Fig. 1. The incidence of MINS was comparable between the two data sets. Table 1 showed no significant differences between the cohorts’ baseline data and preoperative and intraoperative clinical indicators. Although slight numerical differences were observed in BMI categories and the proportion of patients with preoperative hs-cTnT ≥ 14 ng/L between the training and validation cohorts, these differences did not reach statistical significance (*P* = 0.072 and *P* = 0.084, respectively). Given the lack of statistical significance, these minor imbalances are unlikely to have materially influenced the subsequent model validation.

**Table 1:** Baseline characteristics of patient inclusion into the study

Variables	Overall (*n* = 358)	Training cohort (*n* = 251)	validation cohort (*n* = 107)	*P* Value
MINS				0.713
Yes	54 (15.1)	39 (15.5)	15 (14.0)	
No	304 (84.9)	212 (84.5)	92 (86.0)	
Gender				0.969
Male	232 (64.8)	162 (64.5)	70 (65.4)	
Female	126 (35.2)	89 (35.5)	37 (34.6)	
Age (yr)	67.4 ± 8.3	67.4 ± 8.5	67.4 ± 8.0	0.939
Body mass index (kg/m^2^)				0.072
<18.5	17 (4.7)	9 (3.6)	8 (7.5)	
18.5–23.9	195 (54.5)	147 (58.6)	48 (44.9)	
24-27.9	120 (33.5)	77 (30.7)	43 (40.2)	
≥ 28	26 (7.3)	18 (7.2)	8 (7.5)	
Preoperative pulse pressure (mmHg)	53.0 ± 13.1	53.5 ± 13.4	52.0 ± 12.4	0.435
ASA classification				0.215
II	152 (42.5)	113 (45.0)	39 (36.4)	
III	205 (57.3)	137 (54.6)	68 (63.6)	
IV	1 (0.2)	1 (0.4)	0 (0.0)	
NYHA classification				0.582
I	205 (57.3)	148 (59.0)	57 (53.3)	
II	145 (40.5)	98 (39.0)	47 (43.9)	
III	8 (2.2)	5 (2.0)	3 (2.8)	
RCRI				0.838
1	231 (64.5)	162 (64.5)	69 (64.5)	
2	97 (27.1)	67 (26.7)	30 (28.0)	
3	25 (7.0)	19 (7.6)	6 (7.6)	
4	5 (1.4)	3 (1.2)	2 (1.9)	
Comorbidities				
Hypertension	160 (44.7)	105 (41.8)	55 (51.4)	0.121
Diabetes	71 (19.8)	50 (19.9)	21 (19.6)	1.000
Stroke	38 (10.6)	29 (11.6)	9 (8.4)	0.486
Coronary artery disease	49 (13.7)	32 (12.7)	17 (15.9)	0.533
Peripheral artery disease	11 (3.1)	8 (3.2)	3 (2.8)	1.000
COPD	5 (1.4)	5 (2.0)	0 (0.0)	0.328
Hyperlipemia	84 (23.5)	59 (23.5)	25 (23.4)	1.000
Smoking	107 (29.9)	76 (30.3)	31 (29.0)	0.904
Preoperative medications				
Statins	27 (7.5)	17 (6.8)	10 (9.3)	0.532
Anticoagulants	37 (10.3)	23 (9.2)	14 (13.1)	0.354
Beta blocker	28 (7.8)	16 (6.4)	12 (11.2)	0.178
Calcium antagonist	105 (29.3)	67 (26.7)	38 (35.5)	0.121
ACEI/ARB	35 (9.8)	24 (9.6)	11 (10.3)	0.988
Insulin	51 (14.2)	37 (14.7)	14 (13.1)	0.806
Hydragogue	3 (0.8)	2 (0.8)	1 (0.9)	1.000
Preoperative laboratory				
FBG (mmol/L)	5.9 ± 2.2	5.9 ± 2.4	5.9 ± 2.0	0.646
Creatinine (µmol/L)	79.0 (64.0, 92.0)	79.0 (64.0, 93.0)	79.0 (67.0, 87.0)	0.977
Cholinesterase (U/L)	7212.0 ± 1823.6	7147.5 ± 1803.2	7363.4 ± 1870.3	0.314
Albumin (g/L)	38.1 ± 3.6	38.2 ± 4.0	38.6 ± 5.4	0.728
hs-CRP (mg/L)	3.4 (1.4, 8.0)	3.1 (1.3, 8.5)	4.1 (1.5, 8.0)	0.325
Fibrinogen (g/L)	3.6 ± 0.8	3.6 ± 0.8	3.7 ± 0.8	0.970
D-dimer (ng/ml)	173.0(106.0, 306.5)	173.0(101.0, 315.0)	174.0(110.0,287.0)	0.863
Hb (g/L)	114.5 ± 19.6	114.8 ± 18.9	113.6 ± 21.3	0.572
Platelet (×10^9^/L)	274.2 ± 94.0	271.3 ± 94.4	281.1 ± 93.1	0.304
NLR	2.3 (1.6, 3.4)	2.4 (1.7, 3.5)	2.1 (1.6, 3.1)	0.063
PLR	150.1 (112.0, 208.8)	153.4 (113.5, 217.1)	141.0 (109.7,197.5)	0.134
TC (mmol/L)	4.7 ± 1.2	4.7 ± 1.2	4.6 ± 1.1	0.663
Triglyceride (mmol/L)	1.3 (0.9, 1.9)	1.3 (0.9, 1.9)	1.2 (0.9, 1.8)	0.458
HDL-C (mmol/L)	1.2 (1.0, 1.4)	1.2 (1.0, 1.4)	1.2 (1.0, 1.4)	0.703
LDL-C (mmol/L)	2.8 ± 0.9	2.8 ± 0.9	2.8 ± 0.9	0.694
CK (U/L)	91.0 (63.0, 135.0)	90.0 (62.0, 133.0)	95.0 (65.0, 149.0)	0.303
CK-MB (U/L)	16.0 (13.0, 20.0)	15.0 (13.0, 20.0)	17.0 (13.0, 19.0)	0.717
LDH (U/L)	185.0 (163.8, 214.0)	184.0 (163.0, 213.0)	191.0 (166.0, 215.0)	0.436
hs-cTnT (ng/L)				0.084
<14	309 (86.3)	211 (84.1)	98 (91.6)	
≥ 14	49 (13.7)	40 (15.9)	9 (8.4)	
Electrocardiograph				
ST-T changes	78 (21.8)	54 (21.5)	24 (22.4)	0.958
T wave change	39 (10.9)	27 (10.8)	12 (11.2)	1.000
Abnormal q-wave	27 (7.5)	19 (7.6)	8 (7.5)	1.000
HHD				0.981
Yes	75 (20.9)	52 (20.7)	23 (21.5)	
No	283 (79.1)	199 (79.3)	84 (78.5)	
Intraoperative characteristics				
Duration of surgery (min)	161.7 ± 58.3	159.5 ± 58.9	166.7 ± 56.6	0.186
Duration of anesthesia (min)	202.8 ± 60.3	200.7 ± 61.1	207.8 ± 58.4	0.208
Infusion volume (ml)	1763.8 ± 601.3	1760.4 ± 608.5	1771.9 ± 586.8	0.967
Blood Loss (ml)	50.0 (30.0, 80.0)	50.0 (30.0, 85.0)	50.0 (30.0, 70.0)	0.256
SAS	9.0 (9.0, 10.0)	9.0 (9.0, 10.0)	9.0 (8.0, 10.0)	0.255
mSAS	9.0 (8.0, 10.0)	9.0 (8.0, 10.0)	9.0 (8.0, 9.0)	0.201
Tachycardia	23 (6.4)	14 (5.6)	9 (8.4)	0.317
Bradycardia	65 (18.2)	41 (16.3)	24 (22.4)	0.223
Hypertension	141 (39.4)	98 (39.0)	43 (40.2)	0.933
Hypotension	193 (53.9)	132 (52.6)	61 (57.0)	0.514
Transfusion				0.774
Yes	49 (13.7)	33 (13.1)	16 (15.0)	
No	309 (86.3)	218 (86.9)	91 (85.0)	
Vasopressor				0.070
Yes	193 (53.9)	127 (50.6)	66 (61.7)	
No	165 (46.1)	124 (49.4)	41 (38.3)	
Noradrenaline				0.236
Yes	50 (14.0)	31 (12.4)	19 (17.8)	
No	308 (86.0)	220 (87.6)	88 (82.2)	
Postoperative Hb (g/L)	113.4 ± 17.0	113.5 ± 16.5	113.1 ± 18.3	0.828
Types of colorectal surgery				0.790
Dixon surgery of rectal cancer	143 (39.9)	104 (41.4)	39 (36.4)	
Colorectal surgery	97 (27.1)	68 (27.1)	29 (27.1)	
Left hemicolectomy	25 (7.0)	17 (6.8)	8 (7.5)	
Right hemicolectomy	93 (26.0)	62 (24.7)	31 (29.0)	

Data are presented as *n* (%), mean (±standard deviation), or as median [25th percentile - 75th percentile]

*COPD *chronic obstructive pulmonary disease, *ACEI *angiotensin-converting enzyme inhibitor, *ARB *angiotensin receptor blocker, *FBG* fasting blood glucose, *Hb *hemoglobin, *hs-CRP *high-sensitivity C-reactive protein, *NLR *neutrophil to lymphocyte ratio, *PLR *platelet to lymphocyte ratio, *TC *total cholesterol, *HDL-C *high-density lipoprotein cholesterol, *LDL-C *low-density lipoprotein cholesterol, *CK *creatine kinase, *CK-MB *creatine kinase-MB, *LDH *lactate dehydrogenase, *HHD *hypertensive heart disease

### Univariate and multivariate analyses of the training cohort

Sixteen potential risk factors associated with MINS were identified by univariate analysis in the training cohort (Table 2). Before entering these variables into a multivariable logistic regression model, multicollinearity among them was assessed using the variance inflation factor (VIF) and tolerance (TOL). One variable, intraoperative blood transfusion, showed evidence of multicollinearity (TOL = 0.053, VIF = 18.953, exceeding the common thresholds of TOL < 0.1 or VIF > 10) and was therefore excluded from further analysis. The remaining 15 variables were re‑assessed for multicollinearity. All remaining variables had TOL > 0.1 and VIF < 10, indicating no concerning collinearity (Supplementary Table S1). These 15 variables were subsequently entered into a multivariable logistic regression model. Among these, 11 variables were not retained in the final model because they did not reach statistical significance after adjustment. The final model identified age, preoperative hemoglobin, preoperative hs‑cTnT, and SAS as independent predictors of MINS in high‑risk patients undergoing laparoscopic colorectal cancer surgery (Table 3).

**Table 2:** Univariate analysis of MINS in the training cohort

Variables	No MINS (*n* = 212)	MINS (*n* = 39)	*P* Value
Gender			0.079
Male	132 (62.3)	30 (76.9)	
Female	80 (37.7)	9 (23.1)	
Age (yr)	66.8 ± 8.1	70.5 ± 10.0	0.011
Body mass index (kg/m^2^)			0.039
<18.5	6 (2.8)	3 (7.7)	
18.5–23.9	119 (56.1)	28 (71.8)	
24-27.9	69 (32.6)	8 (20.5)	
≥ 28	18 (8.5)	0 (0.0)	
Preoperative pulse pressure	52.7 ± 12.7	57.8 ± 14.9	0.024
ASA classification			0.013
II	101 (47.6)	12 (30.8)	
III	111 (52.4)	26 (66.6)	
IV	0 (0.0)	1 (2.6)	
NYHA classification			0.001
I	133 (62.7)	15 (38.5)	
II	77 (36.3)	21 (53.8)	
III	2 (1.0)	3 (7.7)	
RCRI			0.222
1	142 (67.0)	20 (51.3)	
2	54 (25.5)	13 (33.3)	
3	14 (6.6)	5 (12.8)	
4	2 (0.9)	1 (2.6)	
Comorbidities			
Hypertension	84 (39.6)	21 (53.8)	0.098
Diabetes	42 (19.8)	8 (20.5)	0.920
Stroke	22 (10.3)	7 (17.9)	0.174
Coronary artery disease	23 (10.8)	9 (23.1)	0.065
Peripheral artery disease	6 (2.8)	2 (5.1)	0.799
COPD	4 (1.9)	1 (2.6)	0.573
Hyperlipemia	52 (24.5)	7 (17.9)	0.373
Smoking	62 (29.2)	14 (35.9)	0.406
Preoperative medications			
Statins	12 (5.7)	5 (12.8)	0.197
Anticoagulants	18 (8.5)	5 (12.8)	0.576
Beta blocker	11 (5.2)	5 (12.8)	0.151
Calcium antagonist	53 (25)	14 (35.9)	0.157
ACEI/ARB	21 (9.9)	3 (7.7)	0.892
Insulin	31 (14.6)	6 (15.4)	0.902
Hydragogue	2 (0.9)	0 (0.0)	1.000
Preoperative laboratory			
FBG (mmol/L)	5.8 ± 2.4	5.9 ± 2.0	0.870
Creatinine (µmol/L)	77.5 (63.0, 90.0)	90.0 (73.0, 118.0)	0.005
Cholinesterase (U/L)	7335.3 ± 1690.1	6126.6 ± 2065.2	<0.001
Albumin (g/L)	38.5 ± 4.0	36.9 ± 3.8	0.030
hs-CRP (mg/L)	3.1 (1.3, 8.0)	3.3 (1.2, 15.7)	0.741
Fibrinogen (g/L)	3.6 ± 0.8	3.7 ± 0.8	0.730
D-dimer (ng/ml)	161.5 (100.0, 275.5)	331.0 (166.0, 477.0)	0.001
Hb (g/L)	116.6 ± 18.3	105.4 ± 20.0	0.001
Platelet (×10^9^/L)	271.4 ± 95.8	270.6 ± 88.0	0.960
NLR	2.3 (1.5, 3.1)	2.5 (2.0, 4.2)	0.086
PLR	143.7 (108.8, 197.4)	172.8 (131.8, 250.3)	0.062
TC (mmol/L)	4.8 ± 1.1	4.4 ± 1.3	0.094
Triglyceride (mmol/L)	1.3 (0.9, 2.1)	1.3 (0.7, 1.6)	0.147
HDL-C (mmol/L)	1.2 (1.0, 1.4)	1.2 (1.0, 1.4)	0.645
LDL-C (mmol/L)	2.9 ± 0.9	2.6 ± 0.9	0.054
CK (U/L)	88.0 (62.3, 132.5)	95.0 (60.0, 133.0)	0.778
CK-MB (U/L)	15.0 (13.0, 20.0)	15.0 (12.0, 19.0)	0.607
LDH (U/L)	183.0 (162.0, 210.0)	190.0 (174.0, 214.0)	0.249
hs-cTnT (ng/L)			<0.001
<14	195 (92.0)	16 (41.0)	
≥ 14	17 (8.0)	23 (59.0)	
Electrocardiograph			
ST-T changes	43 (20.3)	11 (28.2)	0.269
T wave change	22 (10.4)	5 (12.8)	0.864
Abnormal q-wave	13 (6.1)	6 (15.4)	0.093
HHD			0.003
Yes	37 (17.5)	15 (38.5)	
No	175 (82.5)	24 (61.5)	
Intraoperative characteristics			
Duration of surgery (min)	159.5 ± 59.1	159.6 ± 58.8	0.996
Duration of anesthesia (min)	200.0 ± 61.4	204.4 ± 59.8	0.677
Infusion volume (ml)	1737.7 ± 568.2	1883.8 ± 790.5	0.169
Blood Loss (ml)	50.0 (30.0, 97.5)	50.0 (30.0, 80.0)	0.866
SAS	9.0 (9.0, 10.0)	9.0 (8.0, 9.0)	0.027
mSAS	9.0 (8.0, 10.0)	9.0 (6.0, 9.0)	0.001
Tachycardia	5 (2.4)	1 (2.6)	1.000
Bradycardia	31 (14.6)	10 (25.6)	0.087
Hypertension	80 (37.7)	18 (41.2)	0.322
Hypotension	110 (51.9)	22 (56.4)	0.603
Transfusion			<0.001
Yes	20 (9.4)	13 (33.3)	
No	192 (90.6)	26 (66.7)	
Vasopressor			0.546
Yes	109 (51.4)	18 (46.2)	
No	103 (48.6)	21 (53.8)	
Noradrenaline			0.092
Yes	23 (10.8)	8 (20.5)	
No	189 (89.2)	31 (79.5)	
Postoperative Hb (g/L)	115.0 ± 16.0	105.6 ± 17.3	0.001
Types of colorectal surgery			0.752
Dixon surgery of rectal cancer	87 (41.0)	17 (43.6)	
Colorectal surgery	59 (27.8)	9 (23.1)	
Left hemicolectomy	13 (6.2)	4 (10.3)	
Right hemicolectomy	53 (25.0)	9 (23.1)	

Data are presented as *n* (%), mean (± standard deviation), or as median [25th percentile − 75th percentile]

**Table 3:** Multivariate analysis of MINS in the training cohort

Variables	Beta coefficient	OR (95%CI)	*P*-value
Age	0.165	1.180 (1.063, 1.309)	0.002
Pre-operative laboratory			
Hb	-0.093	0.911 (0.867, 0.957)	<0.001
hs-cTnT	2.489	12.044 (2.848, 50.945)	0.001
Intraoperative characteristics			
SAS	-0.966	0.380 (0.149, 0.968)	0.043

*OR* odds ratio, *CI *confidence interval

### Development of a dynamic nomogram

**Fig. 2:**
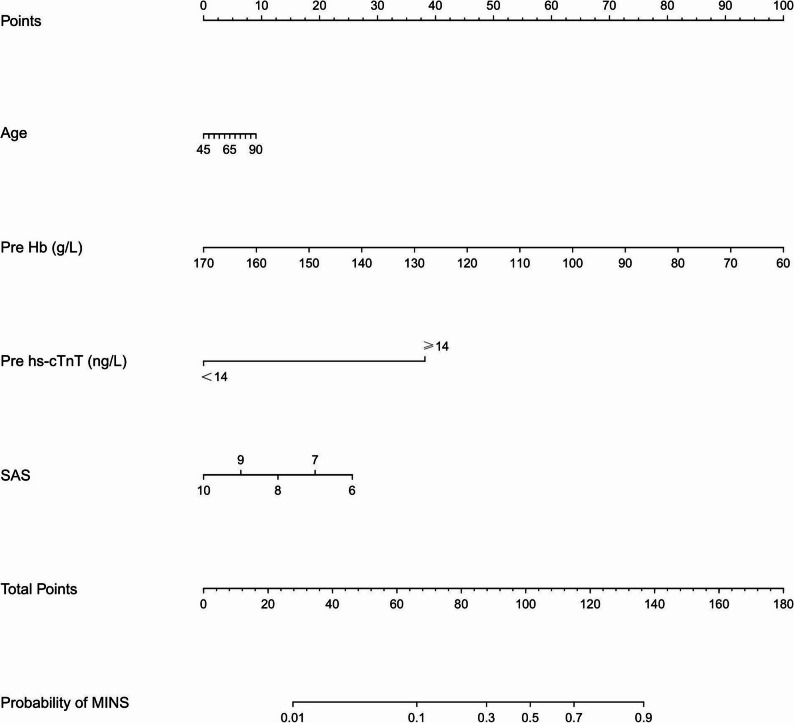
Nomogram clinical prediction model for the risk of MINS (Pre Hb, Preoperative Hemoglobin; Pre hs-cTnT, Preoperative hs-cTnT)

A nomogram was developed based on the results of a multivariate logistic regression analysis with four independent predictors (age, preoperative Hb, preoperative hs-cTnT, and SAS) (Fig. 2). The nomogram was used as follows: the sum of the scores corresponding to each variable was the total score. The predicted risk corresponding to the total score was the risk probability of developing MINS. To facilitate the clinical use of this Nomogram for risk assessment of surgical patients, we developed a dynamic web-based Nomogram (https://pro-of-mins.shinyapps.io/DynNomapp/) to calculate the risk probability of developing MINS in high-risk patients undergoing laparoscopic colorectal cancer surgery.

### Evaluation and internal validation of the dynamic nomogram

The nomogram was evaluated and internally validated by measuring discrimination, calibration, and clinical validity in both the training and validation cohorts.

### Discrimination

**Fig. 3:**
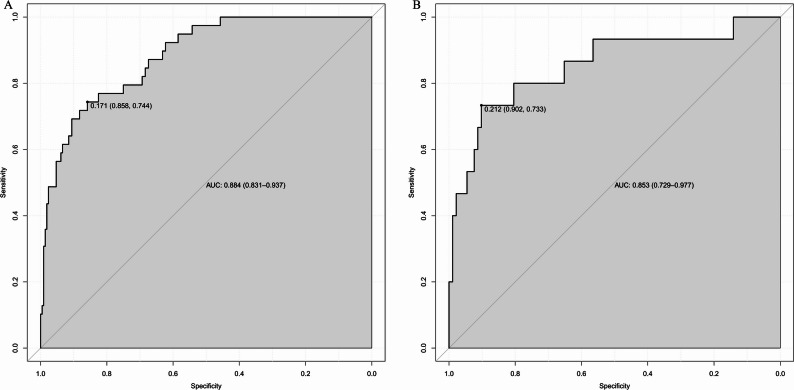
The ROC curve of prediction model (**A**: Training cohort **B**: Validation cohort)

The AUC (C-index) of the nomogram in the training cohort was 0.884 (95% CI: 0.831–0.937), with the cut-off value maximizing the Youden index being 17.1%, which yielded a sensitivity of 74.4%, a specificity of 85.8%, and a corresponding Youden index of 60.2% (Fig. 3A). In the validation cohort, the AUC was 0.853 (95% CI: 0.729–0.977), with the Youden-index cut-off of 21.2% giving a sensitivity of 73.3%, a specificity of 90.2%, and a Youden index of 63.5% (Fig. 3B). Both AUC values exceeded 0.75, a threshold commonly used to indicate acceptable discriminative ability [23].

### Calibration

**Fig. 4:**
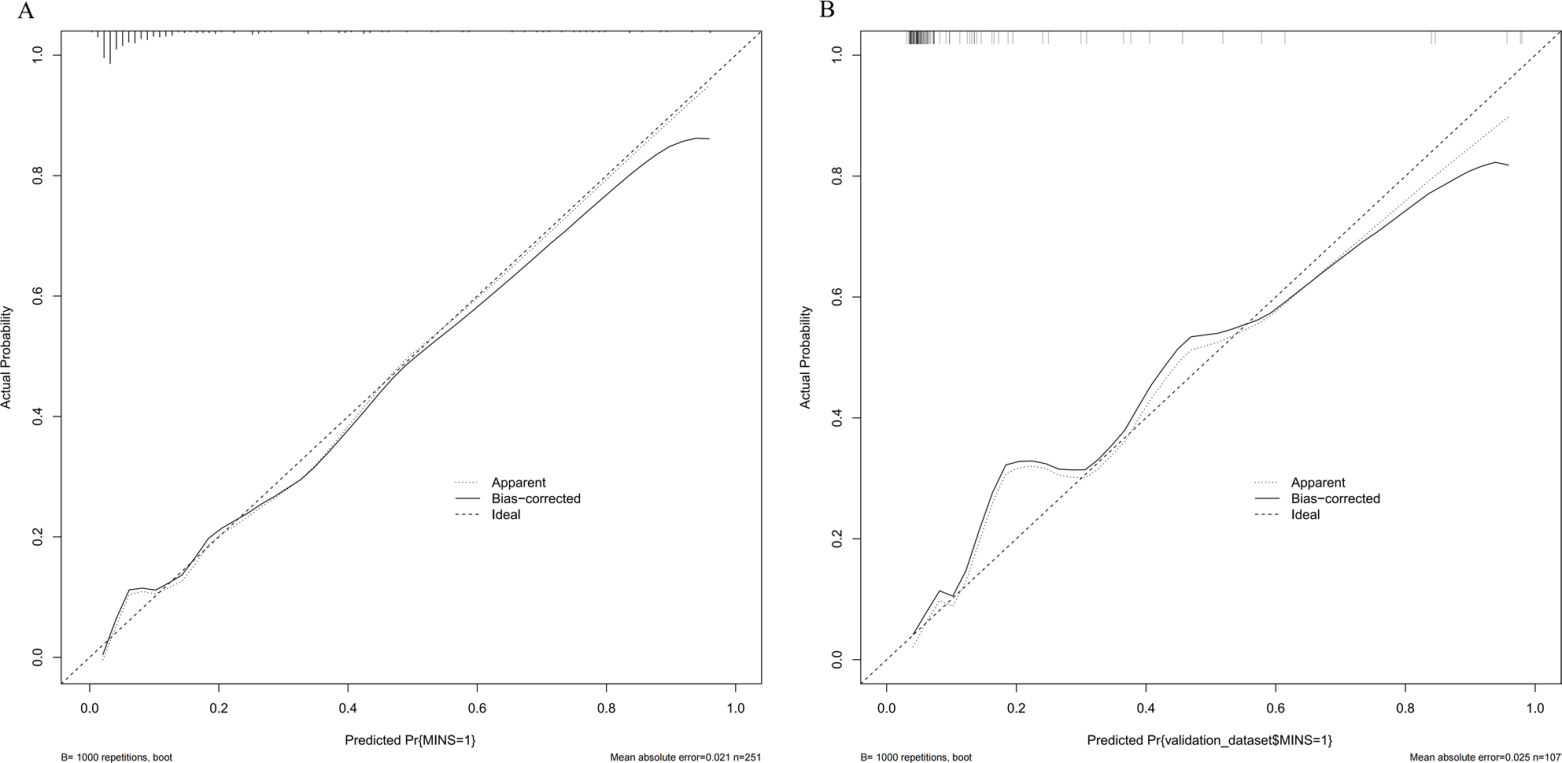
The calibration plot of prediction model (**A**: Training cohort **B**: Validation cohort)

The closer the calibration curve is to the diagonal dashed line, the closer the predicted probability is to the actual probability of occurrence. In the training cohort, the calibration curve demonstrated good agreement between predicted and actual probabilities, with a mean absolute error of 0.021 (Fig. 4A). The Hosmer‑Lemeshow test yielded a χ² value of 9.103 (*P* = 0.334), suggesting adequate calibration. In the validation cohort, the calibration curve also showed good alignment (mean absolute error 0.025, Fig. 4B). The Hosmer‑Lemeshow test confirmed adequate calibration, with χ² = 8.127 and *P* = 0.421. These findings suggest that the nomogram demonstrates satisfactory calibration in both cohorts.

### Decision curve analysis

**Fig. 5:**
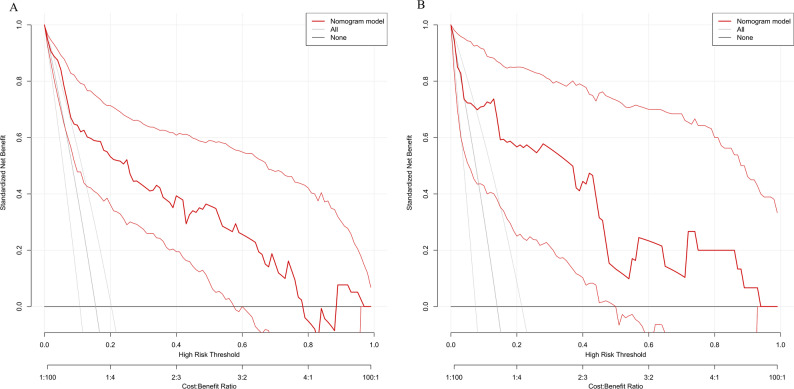
The DCA diagram of prediction model (**A**: Training cohort **B: **Validation cohort)

According to the decision curve analysis, the threshold probability of the training cohort ranges from 3.0% to 78.0%, corresponding to a net benefit of 0 to 87.0% (Fig. 5A). The predictive model also performed well in the validation cohort, with threshold probabilities ranging from 6.0% to 93.0%, corresponding to a net benefit of 0 to 72.0% (Fig. 5B). The results of DCA indicated that the overall net benefit of this nomogram prediction model was favorable, suggesting potential clinical utility in supporting decision-making.

### Performance at the 20% risk threshold

The optimal cut-offs were 17.1% and 21.2% in the training and validation cohorts, respectively. Decision curve analysis confirmed a positive net benefit across a wide range of threshold probabilities, including values between 17% and 22%. To provide a single, clinically intuitive threshold for routine postoperative triage, a risk cut-off of 20% was selected. At this threshold, the nomogram achieved a sensitivity of 69.2%, specificity of 83.0%, positive predictive value (PPV) of 42.9%, and negative predictive value (NPV) of 93.6% in the training cohort; in the validation cohort, the sensitivity was 80.0%, specificity 77.2%, PPV 36.4%, and NPV 95.9%. In the pooled cohort, the sensitivity, specificity, PPV, and NPV were 72.2%, 81.2%, 40.6%, and 94.3%, respectively. Detailed performance metrics for each cohort are shown in Table 4.

**Table 4:** Performance of the nomogram at the 20% risk threshold

Cohort	Sensitivity (%)	Specificity (%)	PPV (%)	NPV (%)
Training cohort	69.2	83.0	42.9	93.6
Validation cohort	80.0	77.2	36.4	95.9
Pooled cohort	72.2	81.2	40.6	94.3

*PPV *positive predictive value, *NPV *negative predictive value

### Comparison with the Revised Cardiac Risk Index

To directly compare the predictive performance of our nomogram with that of the RCRI, we assessed the discriminative ability of the RCRI score for MINS in the validation cohort (*n* = 107) using ROC analysis. The RCRI yielded an AUC of 0.541 (95% CI 0.380–0.702), suggesting limited discriminatory power for MINS in this cohort. In contrast, our nomogram achieved an AUC of 0.853 (95% CI 0.729–0.977) in the same validation cohort, which was significantly higher (DeLong test, *P* < 0.001).

## Discussion

In this study, we developed and validated a postoperative dynamic nomogram for predicting the occurrence of MINS in patients at high risk for colorectal cancer surgery. The nomogram was based on easily accessible clinical variables assessed preoperatively and incorporated intraoperative variables to improve the predictive and discriminatory power of the model. It included age, preoperative Hb, preoperative hs-cTnT, and SAS. The inclusion of the SAS, while precluding preoperative application, enables a more accurate assessment of MINS risk by incorporating real-time intraoperative hemodynamic data. This trade-off reflects the model’s intended purpose: to stratify patients immediately after surgery for targeted troponin monitoring. With favorable discrimination and calibration, the model may be used immediately after surgery to assist clinicians in identifying patients at differential risk for MINS, thereby guiding cTn monitoring and individualized intervention.

Our model does not aim to replace preoperative risk assessment but rather to calibrate and refine it by integrating real‑time intraoperative hemodynamic data. For example, an elderly patient (≥ 65 years) with low preoperative risk according to traditional indices, such as the Revised Cardiac Risk Index (RCRI = 0), may be reclassified as high‑risk if they experience significant intraoperative hypotension or hemodynamic instability (as reflected by a low SAS), thereby warranting postoperative troponin monitoring. Conversely, a patient with high preoperative cardiovascular risk (e.g., history of coronary artery disease) who undergoes an uneventful surgery with stable intraoperative parameters may be downgraded to low‑risk, potentially avoiding unnecessary troponin testing. This re‑stratification is precisely the added value of incorporating intraoperative data, as supported by recent studies demonstrating that the SAS significantly improves the predictive performance of preoperative risk indices [24]. Thus, the primary utility of our model lies in postoperative triage of monitoring resources, identifying patients who will benefit most from troponin screening, while also indirectly encouraging early consideration of hemodynamic optimization during surgery.

In perioperative cardiac risk assessment, the older the patient, the higher the risk of adverse cardiovascular events [25]. The VISION prospective cohort study found that age ≥ 75 years was an independent predictor of MINS [26]. The results of this study also suggest that age was a preoperative independent predictor of MINS. In our training cohort, patients who developed MINS were older (70.5 ± 10.0 years) than those without MINS (66.8 ± 8.1 years). With increasing age, patients’ reserve capacity and compensatory capacity gradually decrease, and the more preoperative comorbidities of underlying diseases, the poorer their tolerance to surgery and anesthesia, and the more likely they are to develop MINS postoperatively.

A mismatch between oxygen supply and demand has been suggested as one of the underlying mechanisms of MINS [27]. In low Hb levels, the heart must maintain a high per-pulse output and heart rate to sustain adequate systemic oxygen delivery, thereby increasing myocardial oxygen demand [28]. Anemia in combination with hypotension, tachycardia, and the use of perioperative positive inotropic drugs may exacerbate myocardial oxygen supply-demand imbalance, which may lead to myocardial injury or type II myocardial infarction [10]. Kwon et al. found that preoperative anemia was independently associated with MINS and that there was a graded association between anemia severity and MINS [29]. The results of the present study also showed that the incidence of MINS increased with decreasing preoperative Hb levels. Specifically, in our training cohort, patients who developed MINS had significantly lower preoperative hemoglobin (105.4 ± 20.0 g/L) than those without MINS (116.6 ± 18.3 g/L). Therefore, preoperative low Hb levels may warrant optimization to help reduce the risk of MINS.

Preoperative high-sensitivity troponin levels contain important prognostic information [9]. A meta-analysis including 10,371 patients showed that preoperative troponin elevation was significantly associated with postoperative MACE and mortality in patients who underwent noncardiac surgery [30]. Chew et al. demonstrated that patients undergoing elective noncardiac surgery with preoperative hs-cTnT ≥ 14 ng/L had the best threshold for predicting major adverse cardiovascular events [31]. In the context of our research, a notable augmentation in the incidence of MINS was observed when the preoperative levels of hs-cTnT were elevated. In our training cohort, 59.0% of patients who developed MINS had preoperative hs-cTnT ≥ 14 ng/L, compared to only 8.0% in the non-MINS group. Therefore, the results of this study suggest that routine measurement of high-sensitivity troponin levels in patients with high cardiovascular risk factors before surgery may help decide whether to perform troponin screening after surgery and make early clinical intervention possible.

A recent retrospective study of the association between intraoperative hypotension and adverse outcomes demonstrated a graded association between intraoperative hypotension and postoperative myocardial injury and mortality, with the degree of hypotension having a more significant impact than its duration [32]. The risk of myocardial injury increased progressively when patients had an intraoperative MAP ≤ 65 mmHg [33]. The results of a prospective VISION study of patients undergoing non-cardiac surgery also showed that an HR of more than 100 beats/min and a maximum SBP > 160 mmHg were associated with an increased risk of MINS, whereas a minimum intraoperative HR of < 55 beats/min was associated with a reduced risk of MINS and mortality [34]. In addition, Park et al. showed that the incidence of MINS was related to intraoperative blood loss, and the threshold intraoperative hemoglobin level associated with MINS was 99 g/L. Maintaining an appropriate hemoglobin level intraoperatively might help to prevent MINS [35]. Surgical Apgar score (SAS), a novel risk assessment tool consisting of three intraoperative parameters, namely estimated minimum MAP, minimum HR, and blood loss, is easily obtained with current monitoring devices, reflects the patient’s intraoperative hemodynamic stability, and has been shown to predict major complications and mortality after surgery [18]. It has been reported that low SAS might be associated with elevated cTn after noncardiac surgery, and risk stratification based on SAS could guide perioperative cTn monitoring [36]. A retrospective study found that SAS, when combined with the standard preoperative risk index RCRI, improved the accuracy of predicting acute myocardial injury after noncardiac surgery [24]. The results of this study showed that the smoother the intraoperative hemodynamics, the higher the SAS and the lower the incidence of MINS. In our training cohort, the median SAS was lower in patients who developed MINS (9.0; IQR 8.0–9.0) than in those without MINS (9.0; IQR 9.0–10.0), reflecting less stable intraoperative hemodynamics in the MINS group. It can be seen that the prevention of MINS may depend on the management of patients during the operation. Maintaining intraoperative hemodynamic stability is a key modifiable factor in reducing the risk of MINS [37]. Since the prediction model we constructed includes intraoperative SAS, it may be used to guide postoperative cardiovascular risk stratification through new information obtained at the end of surgery and update the preoperative assessment of patients’ postoperative cardiac risk, thereby better realizing the individualization of perioperative cTn monitoring. Notably, we also evaluated the modified SAS (mSAS), which incorporates intraoperative transfusion into the blood loss component [19]. However, given the minimal blood loss (median 50 mL) and consequently low transfusion rate in our laparoscopic colorectal surgery cohort, mSAS was not retained in the multivariable model, as it added no predictive value over the standard SAS. This suggests that in procedures with minimal bleeding, the original SAS sufficiently captures intraoperative hemodynamic stability. For surgeries with substantial blood loss, the mSAS might offer additional prognostic information; future studies should validate its utility in such settings.

In our study, perioperative hs‑cTnT elevation was precisely defined as the peak postoperative value within the first three days after surgery minus the preoperative baseline value. This approach, aligned with the 2021 AHA scientific statement [20] and recent expert consensus [9], ensures that acute myocardial injury is distinguished from chronic troponin elevations due to pre‑existing cardiac conditions. The importance of a preoperative baseline has been emphasized in contemporary reviews, as it allows identification of acute perioperative changes that carry independent prognostic value beyond isolated postoperative measurements [38]. Compared to the definition used in the VISION study, which primarily relied on a single postoperative threshold of ≥ 20 ng/L with a high‑sensitivity assay, our composite criterion, incorporating both absolute and relative changes, offers the advantage of capturing acute rises from a low baseline while maintaining prognostic relevance [19]. This aligns with the approach used in the BASEL‑PMI study, which demonstrated that even small absolute increases (≥ 14 ng/L) are associated with increased mortality [16]. However, the requirement for a preoperative measurement may limit its applicability in settings where routine preoperative troponin testing is not performed.

The high-risk inclusion criteria used in our study, namely age ≥ 65 years or age ≥ 45 years with preexisting coronary artery disease, peripheral artery disease, or stroke, were originally proposed and validated by Puelacher et al. [16]. In that cohort, the incidence of perioperative myocardial injury was 16% (95% CI 14–17%), approximately twice the rate reported in broader surgical populations using comparable troponin thresholds (e.g., 8% in the VISION study [26]). The consistency of our MINS incidence (15.1%) with these prior reports further supports the validity of our cohort and the comparability of our findings. This definition has been widely adopted in subsequent MINS-related studies to identify patient groups with a significantly increased risk of postoperative myocardial injury compared to unselected surgical cohorts, confirming its generalizability [16, 39–41]. The Puelacher criteria, by directly targeting the population at risk for MINS, offer a more specific and prognostically relevant approach to perioperative risk stratification. In contrast, traditional cardiac risk indices, such as the RCRI, were primarily developed to predict myocardial infarction and cardiac death and have consistently demonstrated limited discriminative ability for MINS (AUC 0.62–0.65) [12, 14, 42]. Vasireddi et al. demonstrated that preoperative risk stratification by RCRI failed to correlate with postoperative troponin elevation or 1-year mortality, and that MINS in preoperatively “low risk” patients portended a 9.6-fold increased risk of 1-year mortality [13]. Consistent with this evidence, RCRI was not significantly associated with MINS in our univariate analysis (*P* = 0.222). Furthermore, our nomogram demonstrated significantly higher discrimination than the traditional RCRI (AUC 0.853 vs. 0.541, *P* < 0.001), highlighting the added value of incorporating intraoperative hemodynamic parameters. Beyond improving discrimination, the inclusion of SAS also enables reclassification of patients who would be misclassified by preoperative risk indices alone. As demonstrated by Daza et al. [24], the SAS enables correct reclassification of patients classified as high-risk by the RCRI who ultimately do not sustain myocardial injury, thereby avoiding unnecessary troponin testing and potentially reducing healthcare costs. This re‑stratification capacity underscores the clinical utility of incorporating intraoperative data into postoperative risk assessment.

The developed dynamic nomogram provides a simple and practical quantitative tool for predicting the risk of developing MINS in patients at high risk for colorectal cancer surgery. The selection of a single, pragmatic risk threshold is critical for clinical translation. The Youden index values of 60.2% and 63.5% in the training and validation cohorts, respectively, further confirm the robustness of the nomogram at its optimal cut‑offs. These values align with the AUCs exceeding 0.85 in both cohorts and support the selection of a pragmatic 20% threshold for clinical use. Based on the Youden index, the decision curve analysis, and the trade-off between sensitivity and specificity, we propose a risk threshold of 20% for triggering postoperative troponin monitoring. It is clinically intuitive that a predicted risk exceeding 20% would prompt clinicians to consider postoperative interventions, such as enhanced monitoring or cardiology consultation [24]. At this threshold, our model maintains a favorable balance between sensitivity (72.2%) and specificity (81.2%) in the pooled cohort, with a high negative predictive value (94.3%) that effectively identifies low-risk patients, thereby optimizing resource allocation. Importantly, DCA demonstrated that the model can provide a positive net benefit at this threshold in both cohorts, supporting its potential clinical utility.

From a clinical perspective, our web-based dynamic nomogram offers several practical advantages. It can serve as a simple, immediately available risk stratification tool at the end of surgery to guide decisions on postoperative troponin monitoring. All constituent variables, namely age, preoperative hemoglobin, preoperative hs‑cTnT, and the Surgical Apgar Score, are routinely recorded or easily measured, ensuring feasibility in daily clinical practice without additional cost. Moreover, by quantifying the contribution of intraoperative hemodynamic stability (SAS) to MINS risk, the model may heighten anesthesiologists’ awareness of modifiable intraoperative factors and indirectly encourage strategies to minimize hypotension, tachycardia, and blood loss. Crucially, the primary clinical value of our model may lie in enabling personalized postoperative monitoring, identifying patients who truly require troponin monitoring while safely excluding those who do not. The SAS could help refine postoperative decision-making by pinpointing patients who would not benefit from routine monitoring. Given that universal troponin screening of all moderate- to high-risk patients incurs substantial costs (e.g., estimated at $56.1 million CAD annually in Canada [43]), our tool offers a cost-effective strategy to target monitoring resources to those at the highest risk, thereby improving healthcare efficiency without compromising patient safety.

The translation of our dynamic nomogram into routine practice will require addressing several real-world implementation challenges, some of which stem from inherent trade-offs of incorporating intraoperative data. First, because the model relies on the SAS, which requires intraoperative hemodynamic information, it cannot be used for preoperative risk stratification, limiting its utility to postoperative triage. Second, its accuracy depends on the completeness and reliability of intraoperative recordings; missing or low-quality data may preclude SAS calculation or reduce precision. Third, to translate the model into routine practice, seamless integration with electronic health records (EHRs) is essential to enable automatic risk calculation at the end of surgery, thereby avoiding any additional workflow burden for clinicians. Clinician training is also needed to ensure appropriate use of the 20% risk threshold, and model performance should be continuously monitored with periodic updates. Furthermore, formal health economic evaluations are warranted to quantify the potential cost-savings associated with implementing our nomogram, as even modest reductions in unnecessary troponin testing could translate into substantial economic benefits at a population level [24, 42]. Addressing these challenges will be critical for successful adoption and sustainable use of the nomogram in diverse clinical settings.

This study has several limitations. First, this was a single-centre, retrospective study, with the possibility of selection bias or unmeasurable confounders. Despite multivariable adjustment, residual confounding cannot be entirely excluded. Second, postoperative hs-cTnT was measured only twice within the first 72 h. This sampling frequency may have missed MINS events occurring after the last measurement or those with transient elevations, potentially leading to an underestimation of the true incidence of MINS. Third, our analysis was restricted to hs-cTnT. Whether incorporating other cardiac biomarkers (e.g., hs-cTnI, natriuretic peptides) would improve model performance remains unknown and warrants further investigation. Fourth, the number of MINS events in the training cohort was modest (39 events), yielding an events-per-parameter ratio of 9.75 for the four retained predictors. This ratio is only slightly above the conventional threshold of 10, raising a potential risk of model overfitting. Therefore, external validation in a large, prospective, multicenter cohort is warranted to further assess the reliability of the model’s predictive ability before clinical extension of its application.

## Conclusions

In this study, age, preoperative Hb, preoperative hs-cTnT, and SAS were identified as independent predictors of MINS in high-risk patients undergoing laparoscopic colorectal cancer surgery. A postoperative dynamic nomogram incorporating these variables showed favorable discrimination and calibration upon internal validation and demonstrated significantly higher discriminative ability compared with the traditional RCRI. While these findings are encouraging, prospective external validation is necessary to confirm the model’s clinical utility and generalizability before it can be recommended for routine use.

## Supplementary Information

Supplementary Material 1: Table S1. Multicollinearity assessment for candidate predictors. Table S2. Normality test (Kolmogorov–Smirnov) results for key continuous variables in the overall cohort (*N* = 358).

## Data Availability

The datasets used and analyzed during the current study are available from the corresponding author on reasonable request.

## Abbreviations

MINS: Myocardial injury after noncardiac surgery
RCRI: Revised Cardiac Risk Index
hs-cTnT: High-sensitivity cardiac troponin T
SBP: Systolic blood pressure
HR: Heart rate
SAS: Surgical Apgar Score
mSAS: modified Surgical Apgar Score
ROC: Receiver operating characteristic
C-index: Concordance index
DCA: Decision curve analysis
