# Supplementary material for: A postoperative dynamic nomogram for predicting myocardial injury after noncardiac surgery in high-risk patients undergoing laparoscopic colorectal cancer resection
Source: BMC Anesthesiol. 2026 May 13;26:403. doi: 10.1186/s12871-026-03893-x (PMC13339509; doi:10.1186/s12871-026-03893-x)
Supplement: Supplementary file 1 — Supplementary Material 1: Table S1. Multicollinearity assessment for candidate predictors. Table S2. Normality test (Kolmogorov–Smirnov) results for key continuous variables in the overall cohort (N = 358). [file 12871_2026_3893_MOESM1_ESM.docx]

**Supplementary Table S1.** Multicollinearity assessment for candidate predictors

| Variables | Tolerance | VIF |
| --- | --- | --- |
| Age | 0.731 | 1.368 |
| BMI | 0.938 | 1.066 |
| Preoperative pulse pressure | 0.889 | 1.125 |
| ASA classification | 0.664 | 1.554 |
| NYHA classification | 0.692 | 1.445 |
| Preoperative laboratory |  |  |
| Creatinine | 0.744 | 1.344 |
| Cholinesterase | 0.675 | 1.482 |
| Albumin | 0.732 | 1.367 |
| D-dimer | 0.861 | 1.161 |
| Hb | 0.496 | 2.016 |
| hs-cTnT | 0.652 | 1.535 |
| HHD | 0.740 | 1.352 |
| Intraoperative characteristics |  |  |
| SAS | 0.392 | 2.553 |
| mSAS | 0.319 | 3.131 |
| Postoperative Hb | 0.567 | 1.763 |

Tolerance < 0.1 or VIF > 10 indicates potential multicollinearity. All variables included in the multivariable analysis

had VIF < 10, indicating no concerning collinearity.

**Supplementary Table S2.** Normality test (Kolmogorov–Smirnov) results for key continuous variables in the overall cohort (N=358)

| Variables | Kolmogorov–Smirnov D statistic | *P*-value |
| --- | --- | --- |
| Age (yr) | 0.038 | 0.200 |
| Preoperative pulse pressure (mmHg) | 0.073 | 0.059 |
| Preoperative Hb (g/L) | 0.045 | 0.074 |
| Preoperative Creatinine (μmol/L) | 0.154 | 0.000 |
| Preoperative Cholinesterase (U/L) | 0.028 | 0.200 |
| Preoperative Albumin (g/L) | 0.059 | 0.071 |
| Preoperative D-dimer (ng/ml) | 0.364 | 0.000 |
| SAS | 0.256 | 0.000 |
| mSAS | 0.288 | 0.000 |
| Postoperative Hb (g/L) | 0.034 | 0.200 |
